# Implementing whole genome sequencing for foodborne pathogen surveillance: insights and recommendations based on expert experiences

**DOI:** 10.3389/fmicb.2025.1707621

**Published:** 2025-12-18

**Authors:** Roan Pijnacker, Maaike van den Beld, Alexander Ullrich, Pieter-Jan Ceyssens, Dieter van Cauteren, Solveig Jore, Eva Møller Nielsen, Steen Ethelberg, Stefano Morabito, Maren Lanzl, Eelco Franz

**Affiliations:** 1 Center for Infectious Disease Control, National Institute for Public Health and the Environment, Bilthoven, Netherlands; 2 Department of Infectious Disease Epidemiology, Robert Koch Institute, Berlin, Germany; 3 Division of Human Bacterial Diseases, Sciensano, Brussels, Belgium; 4 Scientific Directorate of Epidemiology and Public Health, Sciensano, Brussels, Belgium; 5 Section of Zoonotic and Waterborne Infections, Norwegian Institute of Public Health, Oslo, Norway; 6 Department of Bacteria, Parasites and Fungi, Statens Serum Institut, Copenhagen, Denmark; 7 Department of Infectious Disease Epidemiology and Prevention, Statens Serum Institut, Copenhagen, Denmark; 8 Global Health Section, Department of Public Health, University of Copenhagen, Copenhagen, Denmark; 9 Department of Food Safety, Nutrition and Veterinary Public Health, Istituto Superiore di Sanità, Rome, Italy

**Keywords:** whole genome sequencing, surveillance, public health, genomic epidemiology, foodborne pathogens

## Abstract

Whole-genome sequencing (WGS) is increasingly used as the primary typing method for foodborne disease surveillance. It offers high-resolution cluster analysis, interoperability, and comprehensive pathogen characterization. However, implementing WGS-based foodborne surveillance also poses challenges. This paper outlines these challenges and provides practical recommendations. It requires a business plan that details the financial, technical and human resources needed, since setting up WGS-based surveillance requires substantial initial investments. During the initial phase, the per sample costs of WGS are likely higher than with traditional typing method. However, this will align or even go below that when fully transitioned to WGS-based surveillance because WGS data can be used for multiple purposes such as (sero)typing and antimicrobial and virulence characterization. It is advisable to start with a single pathogen to establish a solid foundation, with the aim of having one institutional sequencing facility. Validating accuracy and consistency of results is crucial before expanding to other pathogens. While cross-disciplinary collaboration has always played an important role in foodborne surveillance, the complexity of WGS results now makes it essential for transforming findings into effective interventions. Despite its challenges, advancements in technology and computation capabilities have made it increasingly accessible, ultimately improving public health surveillance and response.

## Background

The advances in whole genome sequencing (WGS) have significantly enhanced the surveillance and detection of foodborne disease outbreaks (Brown et al., 2019; Li et al., 2021). It has also enhanced our comprehension of the diversity of pathogens in circulation and the macroevolutionary principles of host range. The increased use of WGS is largely driven by the increasing speed and decreasing operational and acquisition costs of high-throughput sequencing, along with advancements in computational speed, data storage and bioinformatics tools for typing. The main advantages of WGS over other typing methods include the high resolution for discerning clusters, the uniform character of sequencing data that increases inter-operability, and the one-size-fits character of the methods that allows to obtain different kind of pathogen characterization information from the sequences (Ford et al., 2018; Jenkins et al., 2019; Nadon et al., 2017). As a result, public health institutes (PHIs) are increasingly adopting WGS as a routine and primary typing method for identifying patient clusters or outbreaks and characterizing antimicrobial resistance and virulence profiles. Moreover, the recent Regulation (EU) 2025/179 on cross-border health threats mandates the submission of WGS data of isolates associated or suspected to be associated with a foodborne outbreak with the aim to foster international and intersectoral detection and investigation of multi-national outbreaks.

However, many PHIs still rely on traditional methods like Multiple-Locus Variable Number Tandem Repeat Analysis (MLVA) or are in the pilot phase of using WGS for surveillance. Global and regional guidelines on integrating WGS into foodborne surveillance have outlined challenges and recommendations for implementing WGS-based surveillance, and typically identify priority pathogens and set broad objectives for integrating WGS into public health strategies (European Centre for Disease Prevention and Control [ECDC], 2019; Nadon et al., 2017; World Health Organization [WHO], 2018). However, the specific experiences and lessons learned can vary significantly depending on the local, national, or regional context in which implementation occurs, and guidelines often do not provide detailed, practical guidance on operational aspects such as laboratory validation, staff training, the stepwise transition from traditional methods to routine WGS use, and the integration of WGS data into existing surveillance workflows. Therefore, this paper provides a cross-section of real-world challenges when implementing WGS-based foodborne disease surveillance in the European context and provides recommendations based on the authors’ experience. This paper is based on the direct experiences of microbiologists and epidemiologists from public health institutes in Belgium, Denmark, Germany, Italy, Norway, and the Netherlands. The primary audience for this manuscript are public health agencies who are implementing WGS-based surveillance for foodborne pathogens; however, the guidance and recommendations provided are also relevant to professionals in related fields, such as food safety and veterinary institutes, and national laboratories who are engaged in similar surveillance activities.

## Starting with WGS-based surveillance

Establishing WGS-based surveillance requires a comprehensive business plan that details the financial, technical and human resources needed. WGS infrastructure requires significant initial investments in sequencing equipment, computational hardware, and software. Additionally, this initiative necessitates either hiring new personnel or retraining existing staff. This includes laboratory personnel for DNA extraction, library preparation, and sequencing; bioinformaticians for developing and implementing analysis methods; microbiologists for genomic data analysis and interpretation; epidemiologists to integrate genomic data with epidemiological information; data scientists for organization of applicable data structures; and IT support for managing computational infrastructure. The resources needed for initiating WGS sequencing vary significantly based on several factors, including choice of sequencing equipment to purchase, extend of desired laboratory automation, presence of an institutionally maintained IT infrastructure, costs of wages in your country, competences of available lab personnel, and the computational and bioinformatic support required. The latter depends on choice of software: in-house developed and maintained open-source pipelines, commercially licensed or freely available web-based software. For context, the combined implementation costs for WGS-based sequencing and typing of *Salmonella*, Shiga toxin-producing *Escherichia coli* and *Shigella* in the Netherlands during 2019–2020 exceeded over €500,000, with approximately one-third allocated to material costs and two-thirds to personnel. This estimate was based on the use of an existing and maintained Illumina sequencer, development and validation of open-source pipelines for assembly and *in silico* serotyping by bioinformaticians and microbiologists in accordance with ISO15189 and ISO16140 standards, the use of a commercially licensed program for cgMLST cluster analysis, training of microbiologists, epidemiologists, and laboratory technicians, and the utilization of an existing institutionally maintained IT infrastructure. Importantly, the costs for transitioning to WGS, including equipment and personnel expenses, can vary substantially over time and between countries. Therefore, a business plan for WGS implementation may be different between countries and institutes. A summary roadmap for implementing WGS as routine typing tool for foodborne disease surveillance is provided in Table 1.

**TABLE 1:** Summary roadmap for implementing WGS as routine typing tool for foodborne disease surveillance.

Phase	Pathogens	Sequencing	Bioinformatics	Surveillance	Quality and accreditation
Start	One or few pathogens with relatively low numbers and limited degree of (genomic) complexity/diversity (i.e., *Listeria*)	Out-sourcing sequencing with an external (commercial) partner	Commercial software like CLC Genomic Workbench or Seqsphere, or web-based platforms like Enterobase	Establish collaboration epidemiology and microbiology for integrated cluster assessment; Focus on cluster-analysis; Organize table-top exercises; No data-integration	Relying on external quality assurance; No diagnostic applications
Transition	Expand number of pathogens, including those with higher (genomics) complexity	Out-sourcing or start small-scale in-house sequencing	Start with own (open source) pipelines for quality control, assembly, and characterization (serotyping)	Coupling of epidemiological information with sequences, mostly manual; Develop algorithm for cluster prioritization	Validate characterization pipelines according to your standards, with comparison to traditional typing methods
Advanced	Sequencing as sole and routine characterization and typing tool for all pathogens	In-house high-throughput sequence facility	Full reliance on open-source pipelines for characterization (serotyping, AMR, virulence)	Automated coupling of epidemiological data to sequence data	Automated systems for data-processing and storage; Accredited processes and pipelines can be used for diagnostics

AMR, antimicrobial resistance.

The shortage of trained bioinformaticians and data scientists poses a significant challenge. The complexity of bioinformatics tools and the computational resources required to manage the vast amounts of data generated by WGS, adds to the challenge. Because of these challenges, it is advisable to start with a single pathogen to establish a solid foundation for a WGS-based surveillance system. This approach allows personnel to build expertise, standardize processes, and develop a robust surveillance system before expanding to other pathogens as capacity grows. However, sequencing a relatively small number of isolates can result in higher per-sample costs due to fixed costs if the sequencing runs’ capacity is not fully utilized. Initially, outsourcing sequencing might be more cost-effective, allowing the institute to gain experience and develop bioinformatics and surveillance infrastructure without the immediate challenge of setting up an in-house sequencing facility. On the other hand, sequencing in-house facilitates a deeper understanding of the sequencing process, quality optimization, troubleshooting of generated data, and efficiency improvements. During the initial phase, a steep learning curve can be expected, with rapid improvements in data quality.

Many public health, food safety and veterinary institutes in Europe have already started to perform WGS on at least a few pathogens other than foodborne. We recommend that public health agencies and other relevant stakeholders liaise with colleagues working on other infectious diseases if these have already established WGS for other bacteria or viruses and to join their activities to obtain one institutional sequencing facility.

It is important to recognize that WGS results are not directly comparable to traditional typing methods such as phage-typing, MLVA and pulsed-field gel electrophoresis (PFGE). This is because MLVA types and PFGE profiles cannot be derived from WGS data. This is particularly relevant for pathogens where surveillance information is exchanged between institutes working on human, food or animal health. If one of the institutes uses WGS, while others use traditional typing methods, comparing typing results between human, food or animal isolates will be challenging. Therefore, a parallel shift toward WGS-based typing with partnering institutes is recommended, especially because cross-sectoral collaboration with relevant authorities or institutes in the food and veterinary sector is pivotal for successful outbreak investigations and source tracing of foodborne pathogens. To facilitate consistent surveillance, adjustments to regulatory and legal frameworks are necessary to establish uniform requirements across all institutes and stakeholders. Achieving such harmonization will require a top-down approach, including a financial commitment from governments to support implementation and capacity-building efforts.

## Transitioning to routine WGS-based surveillance

Before fully transitioning to WGS-based surveillance, it is crucial to run WGS-based typing alongside traditional typing to validate accuracy and ensure consistent results. However, it is important to note that WGS may provide different results than traditional typing methods, which can lead to apparent inconsistencies that may not indicate a lack of reliability, but rather reflect the improved discriminatory power of WGS. Therefore, discrepancies observed during the parallel implementation phase should be interpreted with this in mind, and standardization efforts should consider the inherent advances of WGS over traditional typing methods. Although this parallel approach temporarily increases costs during the transition phase, it is necessary and requires institutional and possibly political support to overcome. Participating in organized External Quality Assessments (EQAs) of WGS-based methods can help evaluate whether current standards are met. Once WGS-based surveillance is fully and efficiently employed, per-sample costs will align or even be lower compared to traditional methods because more laboratory automation is possible, WGS data can be used as a “one-size fits all” typing and characterization method for multiple purposes such as (sero)typing, antimicrobial resistance and virulence characterization, but also more targeted outbreak investigations facilitating efficient use of resources. Supplementary Table 1 provides a comparative analysis of the costs associated with traditional typing methods versus whole genome sequencing (WGS) typing in the Netherlands.

After the initial phase, it is recommended to start sequencing more pathogens to have a shorter turn-around time. This is because the sequencer’s batch will be filled more quickly to justify starting a sequencing run, which is designed to sequence batch-wise. Establishing a centralized, more automated sequencing facility for multiple pathogens within your institute, even beyond foodborne pathogens, may therefore help reduce per-sample costs even more. In addition, it is efficient in terms of setting up quality assurance and validation.

If diagnostics based on genomic data are employed and operated under a quality system, the sequencing and data analysis methods should be validated against applicable quality standards (International Organization for Standardization, 2017a,b, 2022). Validation of both wet- and dry-lab components in public health surveillance should adhere to a harmonized quality framework. Wet-lab methodologies should be validated and verified in accordance with ISO 16140 and ISO 15189, thereby ensuring reliability, reproducibility, and traceability of all microbiological assays. For bioinformatic workflows, ISO 16140 applies, in conjunction with the quality management requirements of ISO 15189 and the technical recommendations outlined in ISO 23418, in order to establish robust validation and performance criteria for data analysis pipelines. This integrated approach ensures that both the analytical (wet-lab) and bioinformatic (dry-lab) phases meet equivalent standards of scientific quality, accuracy, and consistency across the entire surveillance workflow. In addition to laboratory accreditation and validation, participation in EQAs and ring trials, such as those regularly organized by the ECDC and European Union Reference Laboratories (EURLs), is strongly recommended. These interlaboratory comparisons provide a practical framework for benchmarking sequencing and analysis workflows, ensuring comparability of results across institutions and countries, and identifying areas for improvement. It is recommended to validate wet-lab and data-analysis processes in blocks to facilitate targeted verification process as reagent kits, tools or databases are updated. Blocks that can be considered are DNA extraction, library preparation and sequencing in wet-lab procedures and *de novo* assembly, (sero)typing, AMR detection, virulence gene detection and cluster distance calculation in data-analysis processes. Additionally, other structures, such as backup sequencing equipment and the validation of multiple reagents kits, should be in place to ensure continuity in case of equipment failure or reagent shortages. A rigorous maintenance schedule for bioinformatic pipelines is also necessary to balance the use of accredited pipelines with the latest tools and databases. The overall validation and verification process requires continuous availability and collaboration between bioinformaticians and microbiologists.

For data analysis, investing in commercially available software or the use of freely available web-based platforms such as Enterobase, Pathogenwatch and Galaxy can be beneficial, particularly for institutions with limited bioinformatics expertise across public health, food safety or veterinary sectors. These solutions are generally user-friendly for non-bioinformaticians and provide good visualizations, helping microbiologists familiarize themselves with the data (Segerman and Skarin, 2024). However, it is important to consider that commercial software may limit transparency, data sharing, flexibility, and interoperability, especially as operations scale up and the need to interact with (inter)national databases and the need for customized workflows increases. Moreover, reliance on commercial software can create vendor dependency, which may lead to acute problems if the software are no longer being maintained or supported, especially when such software is integrated in routine workflow. This can occur, for example, due to bankruptcy or acquisition by another company. As data production increases, transitioning to automated data-analysis becomes advantageous, both to reduce labor intensity and to maintain full control over analytical processes and version updates. Open-source tools tailored to foodborne pathogens are readily available and can be utilized by bioinformaticians to develop custom software packages that meet specific needs (Maljkovic Berry et al., 2020; Uelze et al., 2020). Early adoption of open-source solutions help avoid workflow disruption and redundant training efforts associated with switching platforms later. Where initial adoption of commercial software is necessary due to resource or expertise constraints, we recommend planning for a gradual transition to open-source alternatives as capacity builds, ensuring sustainable and interoperable data analysis practices in the long term.

In the long-run, continued funding is required for maintenance of bioinformatics pipelines, computing infrastructure, but also scaling up with increasing throughput and technological processes. Without continued funding, initial investments in setting up WGS-based surveillance are becoming unsustainable, limiting the long-term impact and utility of surveillance efforts.

## Bridging the gap between microbiology, epidemiology and other disciplines

Cross-disciplinary collaboration has always been important in surveillance of foodborne pathogens. However, given the complexity of WGS interpretation, genomic surveillance necessitates intensified collaboration and mutual understanding between microbiologists and epidemiologists. Bioinformaticians, who traditionally align more closely with microbiologists, can play a crucial role in facilitating this collaboration. It is essential for microbiologists and epidemiologists to work together to understand WGS results, integrate important epidemiological data, and provide genomic and microbial context, in order to make informed public health decisions. The latter also requires the authorities to be part of the information sharing process and it is crucial that they understand the meaning of the WGS results being the final recipients of the information and expected to take actions. Forming a multidisciplinary team within and between institutes and encouraging the exchange of personnel between epidemiology, microbiology and decision makers can enhance mutual understanding and provide valuable training opportunities. Integration of genomic and epidemiological data is also needed to avoid duplicate analyses of data which belong to the same patients. Moreover, combining such data is needed to identify and prioritize clusters of interests in e.g., young age groups, geographically clustered patients, or patients with a common food consumption pattern.

Epidemiological data could be as simple as age, sex, and place of residence of patients, but also more detailed food consumption or traveling data from patient interviews. Patient demographics data can be relatively straight-forward to integrate with WGS data, since these metadata often accompany the isolate from which the sequences are generated. More detailed epidemiological data, however, can be difficult to link to sequence data because there may not be a common patient identifier linking both data sources, but whether this is possible strongly depends on national surveillance systems and legislation. If a common identifier is not available, statistical matching could be used based on common variables such as age, sex and place of residence. In practice, the integration could be done by annotating phylogenetic or minimum spanning trees with epidemiological information, or by adding columns of cluster identifiers (e.g., which patients cluster based on different genetic distances) to epidemiological data.

Regularly conducting outbreak simulations based on WGS data is advisable, as it helps identify process weaknesses, clarify roles and responsibilities, and foster collaboration between different disciplines and sectors. Furthermore, continued training of personnel is required to ensure that expertise is kept with emergence of novel technologies and mobility of personnel, which requires human and financial resources.

## Cluster detection

While WGS greatly enhances the ability to identify outbreak sources compared to traditional methods, additional evidence is always required to establish causality, including epidemiological data showing that cases have been exposed to a specific source (EFSA Panel on Biological Hazards (EFSA Biohaz Panel) et al., 2019). The assumption behind using WGS for outbreak detection is that cases infected by identical or closely related strains are more likely to have a recent common ancestor and share a common source of exposure, such as contaminated food (Besser et al., 2019).

Distinguishing case cluster isolates from sporadic ones has been the long-standing challenge of genomic epidemiological surveillance. Setting appropriate thresholds for genetic relatedness can be challenging due to the complexity of WGS data and the need to balance sensitivity and specificity in outbreak detection and cluster identification. While commercial bioinformatics software often provides default build-in threshold to aid in initial analysis, transitioning to customized or open-source pipelines necessitates the establishment of user-defined thresholds (Uelze et al., 2020). Some international consensus exists for certain pathogens, primarily informed by cumulative experience comparing epidemiologically confirmed and non-confirmed outbreak cases (Octavia et al., 2015). Although predefined thresholds are frequently applied, defining a cluster cut-off based on genetic distance requires prior knowledge on the evolutionary processes shaping the bacterial population, such as mutation and recombination rates. Several methods have been developed to identify optimal genetic distance thresholds for discriminating between outbreak and non-outbreak isolates. For example, previous work employed various hierarchical clustering methods (single, average and complete) and selected the optimal number of clusters based on the consensus of internal validation indices (Coipan et al., 2020). In addition, they also demonstrated that different allele- and SNP-based typing workflows generate clusters with similar compositions. More recently, a modeling framework has been proposed that estimates genetic-distance thresholds for single-strain outbreaks originating from contaminated environmental or food sources, simulating mutation accumulation using outbreak-specific parameters such as pathogen mutation rate and time since contamination based on outbreak-specific features (Duval et al., 2023). Ultimately, defining an appropriate threshold for cluster detection requires understanding of pathogen population diversity and diversification rates (which may be context specific), the extent to which the genetic variation of the pathogen population is captured by surveillance, and the availability of high-quality epidemiological data. This demands accumulated experience, knowledge, and close collaboration among microbiologists, epidemiologists and bioinformaticians.

## Source identification

The ability to match food, animal or environmental strains to human clusters based on genomic similarity offers a significant advantage over traditional, lower-resolution typing methods in identifying outbreak sources. However, genomic similarity does not always match with epidemiological associations. Strains that appear identical based on WGS could be present in multiple sources, with only one being the true cause of an outbreak. Likewise, a genomically diverse cluster can have evolved from a single source where the strain has diversified over time (Gerner-Smidt et al., 2019; Montalbano Di Filippo et al., 2022). Similarly, strains can temporally persist in their reservoir or production environment with no or limited genomic changes, and infecting people with high similar strains over prolonged periods of time, thereby complicating source attribution (Gerner-Smidt et al., 2019; Montalbano Di Filippo et al., 2022). Such temporal persistence (more than 1 year) of strains with no or very limited genomic changes is for example known for *Listeria* monocytogenes present in food production environments under low temperatures and limited growth, but also for *Salmonella* Enteritidis originating from the laying-hen primary production (Gerner-Smidt et al., 2019; Montalbano Di Filippo et al., 2022). As with cluster threshold determination, accurate source identification requires close integration of WGS-data and high-quality epidemiological data. Moreover, a comprehensive understanding of the food chain, based on trace-back and trace-forward investigations during outbreak investigations, is essential for accurate assessment and interpretation of matching strains and precise identification of potential sources.

## Prioritization of clusters

The primary advantage of WGS for pathogen typing is its higher resolution compared to traditional molecular methods as well as the determination of the genetic relatedness of isolates (Brown et al., 2019; Li et al., 2021). WGS allows detection of small and/or diffuse outbreaks with higher confidence, many of which may go unnoticed with traditional subtyping methods. As the number of detected potential outbreaks or clusters increases with the use of WGS, laboratories and public health authorities need to define which clusters to investigate based on available resources. Clusters with a higher number of cases, those clustering in place and/or time, or those with unusual demographic features may be given higher priority. Each pathogen will likely have its own criteria for cluster delineation and priority assignment for public health action, which will evolve with increased experience following WGS implementation. Defining thresholds to investigate clusters is essential for surveillance, but case definitions may deviate from these pre-set thresholds once an outbreak investigation is initiated (Payne et al., 2021).

It may be tempting to focus surveillance efforts on clusters identified by WGS only. However, it remains important to monitor overall trends in number of cases, regardless of WGS clustering. This is because an overall increase may be masked by many smaller clusters and would otherwise go unnoticed.

## Communicating about clusters

Assigning isolates to genetic clusters commonly utilizes distance-based hierarchical clustering based on single nucleotide polymorphism (SNP) approach or alleles from a gene-by-gene approach (Coipan et al., 2020; Pearce et al., 2018; Uelze et al., 2020). Both methods have freely available and commercial software solutions for assembly and visualization. However, communication about clusters can be challenging due to the lack of a universal nomenclature. For example, UK Health Security Agency uses a system of SNP addresses as unique cluster identifiers, requiring the same database to identify new SNP addresses (Elson et al., 2019). The gene-by-gene approach, such as core-genome or whole-genome MLST (cgMLST or wgMLST), uses hierarchical clustering schemes to assign stable cluster group numbers, but this is only feasible within specific databases like Enterobase. These communication challenges are particularly evident in multi-country outbreaks, where multiple systems such as SNP addresses, Enterobase cgMLST hierarchical cluster designations, and specific sequence accession codes are used concurrently (European Centre for Disease Prevention and Control [ECDC], 2024). Recognizing this diversity, we recommend that, particularly for international outbreaks and cross-border surveillance, laboratories should clearly report the cluster definition method and identifier used, and include raw sequence accession numbers (e.g., ENA/NCBI) in communications where possible. Whenever feasible, laboratories should align their cluster communication with the guidelines of the ECDC, as outlined in the “Long-term surveillance framework 2021–2017” (European Centre for Disease Prevention and Control [ECDC], 2023). This framework aims to harmonize nomenclature and data sharing procedures at the European level. Until a universal nomenclature is adopted, we advocate for transparency, dual reporting of method-specific cluster identifiers and accession numbers, and close adherence to ECDC recommendations for cross-border events.

At the same time, multiple studies and organized quality assessments have shown that using different sequencing techniques, platforms, analysis approaches and databases in cluster analysis generally produce highly concordant clusters (Coipan et al., 2020; Mixão et al., 2025; Pearce et al., 2018). For communication within the public health institute and cross-sectoral with partners such as food and veterinary institutes, as well as decision makers, a stable cluster identifier is essential to provide clarity and enable tracking of a cluster over time.

## Data sharing

Sharing sequence data generated by different national institutes, especially between public health, food, and veterinary partners, is important for optimizing source identification of clusters or outbreaks. Ideally, these data would be added to a joint database, or sequence data of specific pathogens and/or serovars or serotypes could be shared if clusters are identified. However, whether this is possible depends on national legislation and the organizational structure of surveillance systems. Sharing all sequence data has the benefit of allowing each institute to independently confirm the other’s analysis, and promotes collaboration between the human, food, and veterinary sectors. Although sharing vast amounts of WGS data can be challenging, an increasing number of data platforms are available for sharing sequence data, including public platforms like Enterobase or ENA, as well as local or cloud-based options (Yuan et al., 2024; Zhou et al., 2020). For sharing sequence data with external partners, such as food authorities, it may be beneficial to share only sequences without or with limited metadata to comply with General Data Protection Regulation (GDPR) regulations in place in the European Union. On a supranational level, the ECDC and the European Food Safety Authority (EFSA) have established fully operational platforms for routine sharing and integration of WGS data sharing from public health and the animal/food sector (European Centre for Disease Prevention and Control [ECDC], and EuropeanFood Safety Authority [EFSA], 2019). These platforms enable streamlined and standardized data uploads, thereby greatly enhancing the capacity for international and intersectoral detection and investigation of multi-national outbreaks.

## Conclusion

Implementing WGS-based surveillance requires careful planning, significant initial financial investments, and strong institutional commitment. Enhanced collaboration among epidemiologists, microbiologists, bioinformaticians, and professionals from other relevant disciplines is essential for effective genomic surveillance. Additionally, coordination with food and animal institutes is crucial to align typing methods and enhance outbreak source identification in a one health framework. EURLs could play a crucial role in establishing and supporting WGS-based surveillance by providing expertise to ensure harmonized implementation across member states. It is important to note that in federal countries, where responsibilities for public health, food safety, and animal health are distributed among multiple entities, coordination at the national level is essential to address internal differences in infrastructure, resources, and expertise before broader harmonization can be achieved. While transitioning to WGS-based surveillance presents challenges, advancement in sequencing technologies and computation capabilities have made it increasingly accessible, ultimately leading to improved public health surveillance and public health response. Additionally, genomic data can aid our understanding of how pathogens evolve, adapt, and spread, which can help developing strategies to combat infectious diseases.

## Data Availability

The original contributions presented in this study are included in this article/Supplementary material, further inquiries can be directed to the corresponding author.
