# Supplementary material for: Implementing whole genome sequencing for foodborne pathogen surveillance: insights and recommendations based on expert experiences
Source: Front Microbiol. 2025 Dec 18;16:1707621. doi: 10.3389/fmicb.2025.1707621 (PMC12756440; doi:10.3389/fmicb.2025.1707621)
Supplement: Supplementary file 1 [file Data_Sheet_1.pdf]

Supplementary table S1. Business case example Salmonella surveillance traditional techniques vs WGS sequencing after implementation of efficient workflows in the Netherlands

Activity/debits per year[1]	Traditional methods	Illumina sequencing	Oxford Nanopore sequencing
**Pre-analysis process, registration and culturing (n=1500)**	**€36,450**	**€36,450**	**€36,450**
○ **Materials**	€3,150	€3,150	€3,150
○ **Personnel**	€33,300	€33,300	€33,300
**Luminex Salmonella serotyping assay (n=1500)**	**€108,375**	**€0**	**€0**
○ Materials	€52,875	€0	€0
○ Personnel	€55,500	€0	€0
**Salmonella serotyping, according to Kaufman and White scheme (n=400)**	**€15,800**	**€0**	**€0**
○ Materials	€1000	€0	€0
○ Personnel	€14,800	€0	€0
**MLVA typing S. Enteritidis/ (mono)Typhimurium (n=900)**	**€22,320**	**€0**	**€0**
○ Materials	€9,000	€0	€0
○ Personnel	€13,320	€0	€0
**PCR Salmonella d-tartrate (mono)Typhimurium (n=300)**	**€4,840**	**€0**	**€0**
○ Materials	€400	€0	€0
○ Personnel	€4440	€0	€0
**Antimicrobial susceptibility tests (n=975)[2]**	**€40,000**	**€0**	**€0**
○ Materials	€20,000	€0	€0
○ Personnel	€20,000	€0	€0
**Illumina sequencing, including automated DNA extraction (n=1500)**	**€0**	**€153,000**	**€0**
○ Materials	€0	€97,500	€0
○ Personnel	€0	€55,500	€0
**Oxford Nanopore sequencing, including automated DNA extraction (n=1500)**	**€0**	**€0**	**€77,250**
○ Materials	€0	€0	€49,500
○ Personnel	€0	€0	€27,750
**Routine WGS data-analysis, incl quality control (n=1500, personnel)**	**€0**	**€19,400**	**€19,400**
**Bioinformatic maintenance and trouble-shooting pipelines/year (personnel)**	**€0**	**€17,760**	**€17,760**
**Computational and data storage costs/year (material)[3]**	**€0[4]**	**€8,500**	**€42,250[5]**
**Total**	**€227,785**	**€235,110**	**€193,110**

Based on a fictive year in the Netherlands with 2025 prices, receiving 1500 Salmonella isolates for surveillance, of which 600 *S.* Enteritidis and 300 *S.* Typimurium (diphasic and monophasic). Traditional methods as applied until 2019 at RIVM, with a Luminex screening for frequently encountered serotypes, followed by slide agglutination for rare serotypes. All *S.* Typhimurium (diphasic and monophasic) were confirmed with PCR (Tennant et al.) and all *S.* Enteritidis and *S.* Typhimurium (diphasic and monophasic) were further typed with MLVA. A total of 65% of all Salmonella isolates were sent to WBVR for antimicrobial susceptibility typing for surveillance purposes only. [1]Excluding equipment depreciation and maintenance. [2]Performed and funded by WBVR. [3]Institutional transfer to IV organization. [4]Neglectable amount. [5]Estimated to be five times higher than with Illumina data.
